# Gene Therapy in Hemophilia: Clinical Advances, Immunological Challenges, and Emerging Therapeutic Perspectives

**DOI:** 10.3390/ijms27093922

**Published:** 2026-04-28

**Authors:** Agata Serrafi, Andrzej Wasilewski, Amelia Wawrzkiewicz, Anna Kałuża, Eliza Wasilewska

**Affiliations:** 1 Department of Biochemistry and Immunochemistry, Division of Chemistry and Immunochemistry, Wroclaw Medical University, M. Skłodowskiej-Curie 48/50, 50-369 Wroclaw, Poland; 2 Student Scientific Association of Medical Chemistry and Immunochemistry, Wroclaw Medical University, M. Skłodowskiej-Curie 48/50, 50-369 Wroclaw, Poland; 3 Department of Allergology, Medical University of Gdansk, 80-214 Gdansk, Poland; 4 Center of Rare Disease, Centre University Hospital, 80-952 Gdansk, Poland

**Keywords:** hemophilia, Adeno-associated virus (AAV) gene therapy, viral vectors, immunogenicity, Roctavian, Hemgenix, post-gene therapy care

## Abstract

Gene therapy is reshaping the therapeutic paradigm in hemophilia by enabling sustained endogenous clotting factor production after a single administration. This approach moves disease management beyond lifelong replacement therapy. While clinical trials have demonstrated marked reductions in bleeding rates and treatment burden, real-world implementation has revealed emerging complexities. These include interindividual variability in transgene expression reflected by a progressive reduction in circulating FVIII or FIX activity over time, uncertainty regarding the long-term durability of expression, immune-mediated constraints, and episodes of transaminase elevation. This review addresses a critical transition point in the field: the shift from proof-of-concept efficacy toward integration of gene therapy into long-term hemophilia care. We examine determinants of therapeutic stability, host–vector immune interactions, and mechanisms underlying loss or fluctuation of expression, with emphasis on monitoring strategies and post-therapy management pathways. Immunogenic processes affecting vector transduction, hepatocellular responses, and transgene persistence are discussed alongside current approaches to immune modulation. This review uniquely focuses on post-gene therapy clinical integration rather than vector design or trial outcomes. Beyond direct factor correction, evolving therapeutic concepts targeting coagulation rebalancing and immune regulation are considered within a systems-based framework. Psychosocial adaptation and patient-reported outcomes are also explored, underscoring that therapeutic success extends beyond hemostatic control. In aggregate, these perspectives position gene therapy not as a singular curative event but as a component of an evolving, biologically integrated management strategy. Long-term follow-up translational research (LTFU) and coordinated global efforts will be essential to optimize durability, safety, and equitable access.

## 1. Introduction

Hemophilia is a hereditary bleeding disorder characterized by deficiency or dysfunction of clotting factor VIII (hemophilia A) or factor IX (hemophilia B) and is historically associated with recurrent bleeding, progressive joint damage, and long-term disability [1,2]. Although regular replacement therapy has transformed hemophilia from a fatal condition into a chronic, manageable disease, treatment remains dependent on lifelong intravenous administration and does not address the underlying genetic defect.

The development of gene therapy has introduced a novel therapeutic paradigm based on sustained endogenous production of the missing clotting factor following a single administration. Clinical studies have demonstrated significant reductions in bleeding frequency and treatment burden, positioning gene therapy as a major advance in hemophilia management [1]. However, long-term durability of expression, interindividual variability in response, and immune-mediated limitations remain important unresolved issues [3].

Most existing reviews have focused primarily on vector design, trial outcomes, and proof-of-concept efficacy [4]. While Herzog et al. comprehensively covered capsid engineering and phase 1/2 efficacy, they devoted minimal attention to the 5–10 year post-infusion management questions that now dominate clinical practice [5]. In contrast, the clinical integration of gene therapy into long-term hemophilia care—including management of variable responses, durability monitoring, immune-related complications, and post-therapy treatment pathways—remains less comprehensively addressed. As gene therapy transitions from clinical trials to routine practice, these aspects are becoming increasingly relevant to patient management [6,7].

This narrative review was based on literature retrieved from PubMed, Scopus, and Google Scholar using predefined keywords related to gene therapy and hemophilia (e.g., “hemophilia gene therapy”, “factor VIII”, “factor IX”, “AAV vectors”, “Roctavian”, “Hemgenix”, “long-term outcomes”, and “immune response”). The search was limited to publications from 2021 to 2025. All article types, including original studies, preclinical studies, clinical trials, and review articles, were considered based on titles and abstracts.

This review therefore provides a clinically oriented and translational perspective on gene therapy in hemophilia, focusing on its real-world clinical integration and strategies for post-therapy management. In addition, we discuss quality-of-life outcomes, psychosocial adaptation, and emerging therapeutic concepts extending beyond direct factor replacement, placing gene therapy within the evolving framework of hemophilia care.

## 2. Gene Therapy in Hemophilia

### 2.1. Mechanisms of AAV Gene Therapy in Hemophilia

Gene therapy for hemophilia is based on liver-directed delivery of a functional factor VIII (FVIII) or factor IX (FIX) gene to enable endogenous production of the missing clotting factor [8,9]. Current clinical strategies primarily employ adeno-associated viral (AAV) vectors due to their hepatotropism, favorable safety profile, and ability to mediate long-term transgene expression [10,11,12].

**Figure 1:**
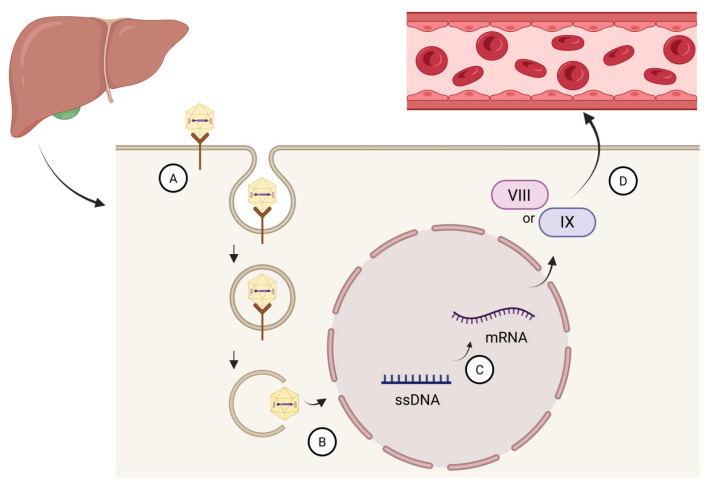
Conceptual framework of AAV-mediated gene therapy in hemophilia. (A) Binding of the adeno-associated viral (AAV) vector to the hepatocyte surface receptor and cellular uptake of the vector. (B) Intracellular trafficking of the AAV vector and delivery of the vector genome to the nucleus. (C) After entry into the nucleus, the single-stranded AAV genome undergoes second-strand synthesis to form double-stranded DNA. (D) Transcription and translation of the therapeutic transgene lead to intracellular production of clotting factor VIII or IX, followed by its secretion into the circulation, restoring hemostasis.

Following systemic administration, AAV vectors bind to specific cell surface receptors and are internalized by hepatocytes. After endosomal escape, the vector genome is transported to the nucleus, where it persists predominantly as episomal DNA rather than integrating into the host genome. This episomal persistence supports sustained transcription of the therapeutic transgene while minimizing the risk of insertional mutagenesis (Figure 1) [10,12,13].

### 2.2. Vector Design Considerations

Vector design is a critical determinant of therapeutic performance. Expression cassettes include liver-specific promoters to enhance hepatocyte-restricted transcription and reduce off-target expression. Codon optimization and regulatory elements are incorporated to improve transcriptional efficiency and protein production [14]. In hemophilia A, the use of B-domain-deleted FVIII constructs enables packaging within the limited AAV genome capacity, whereas hemophilia B gene therapy often utilizes high-activity FIX variants (e.g., FIX-Padua) to enhance functional output at lower expression levels [15,16].

### 2.3. Dose–Response and Determinants of Expression

Dose–response relationships are a central feature of AAV-mediated gene transfer. Higher vector doses generally correlate with increased transgene expression but are also associated with a greater risk of immune-mediated hepatocellular responses. Consequently, therapeutic dosing represents a balance between achieving sufficient factor activity and minimizing immunogenicity [17].

AAV vectors do not support efficient re-administration due to the development of anti-capsid neutralizing antibodies. Therefore, current treatment paradigms rely on single-dose strategies and emphasize patient selection based on pre-existing immunity. Long-term expression durability depends on hepatocyte stability, immune tolerance, and transcriptional regulation of the episomal transgene [18,19]. This platform-based framework underpins current clinical gene therapy approaches in hemophilia and informs ongoing efforts to optimize vector design, dosing strategies, and immune management.

## 3. Immunological Determinants of Gene Therapy Outcomes

### 3.1. Immune Barriers to AAV-Mediated Gene Transfer

Immunogenicity is a major determinant of the efficacy and durability of AAV-mediated gene therapy in hemophilia. Immune responses may target the viral capsid, transduced hepatocytes, or the transgene product, affecting both initial transduction and long-term expression [20,21,22].

### 3.2. Adaptive Immune Responses and Loss of Expression

Pre-existing anti-AAV neutralizing antibodies (NAbs) reduce vector entry into hepatocytes and represent a key eligibility limitation. Post-infusion adaptive immune responses can be broadly divided into two mechanistically distinct components: capsid-specific T cell responses and transgene-specific T cell responses. Capsid-specific T cell responses arise when peptides derived from the viral vector capsid are processed and presented on MHC class I molecules by transduced hepatocytes shortly after vector administration. Activated CD8^+^ T cells recognize these capsid-derived epitopes and eliminate transduced hepatocytes, leading to increased serum aminotransferase activity and a subsequent decrease in transgene expression, whereas transgene-specific T cell responses are directed against peptides derived from the therapeutic protein encoded by the transgene. Transgene-specific CD8^+^ T cells can attack and destroy cells expressing the therapeutic protein, but the response occurs later and is more persistent [5]. In addition to these adaptive immune mechanisms, innate immune components such as Kupffer cells contribute to early vector detection and antigen presentation, while regulatory T cells (Tregs) can modulate immune tolerance and influence the persistence of transgene expression [23]. Therefore, early detection and immunomodulatory intervention are essential to preserve expression [22].

Immune responses directed against the transgene product (FVIII/FIX) are less frequent than with exogenous replacement therapy but remain clinically relevant, especially in patients with limited prior immune tolerance.

**Figure 2:**
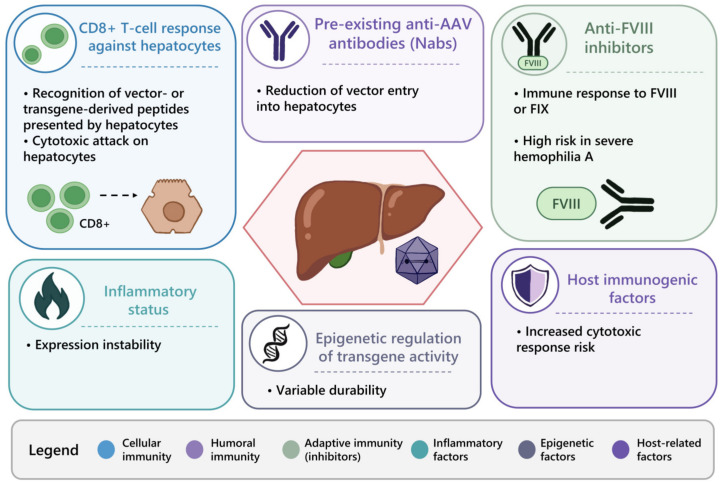
Key immunological barriers in hemophilia gene therapy. The figure summarizes the main factors influencing gene therapy outcomes, including the CD8+ T cell response to transduced hepatocytes, preexisting anti-AAV antibodies limiting vector delivery, and the development of anti-FVIII inhibitors. Additional factors, such as inflammatory status, epigenetic regulation, and host immunogenetic factors, contribute to the variability and persistence of transgene expression.

Long-term expression stability may also be influenced by host immunogenetic factors, inflammatory status, and epigenetic regulation of transgene activity, which together shape the risk of immune-mediated expression decline and are reflected in clinically observable patterns (Figure 2) [24]. These mechanisms provide a conceptual framework for interpreting post-therapy fluctuations in factor activity and guiding clinical monitoring.

### 3.3. Clinical Monitoring and Immunomodulatory Strategies

Immunological determinants of gene therapy outcomes should be considered within a dynamic host–vector interaction framework. Monitoring markers include anti-AAV NAb titers, liver enzyme levels, T-cell activation profiles, and inhibitor testing. Management strategies focus on immunomodulation, most commonly corticosteroids, to prevent immune-mediated loss of expression. Key mechanisms, clinical markers, and management approaches are summarized in Table 1. Further translational research is required to optimize immune monitoring, refine patient selection, and improve vector design to enhance durability and safety.

**Table 1:** Immunological determinants of gene therapy outcomes in hemophilia and corresponding clinical management strategies.

Immune Mechanism	Clinical Markers	Consequence	Clinical Management	References
Pre-existing anti-AAV antibodies	NAb low titers (1:5; LBR) → treatment; NAb higher titers (1:20; HBR) → ineligibility	Poor vector transduction	Patient screening; eligibility criteria	[15,25,26,27]
CD8+ T-cell response against hepatocytes	ALT/AST elevation; T-cell activation	Loss of transgene expression	Corticosteroids; calcineurin inhibitors	[15,25,27,28,29]
Immune response to FVIII/FIX	Inhibitor testing	Neutralization of endogenous factor	Monitoring; factor-based management	[15,26]
Immunogenetic susceptibility	HLA genotype (investigational)	Increased cytotoxic response risk	Risk stratification (research phase)	[30]
Inflammatory milieu	Cytokine profiles	Expression instability	Early immunomodulation	[15,27]
Epigenetic regulation	Emerging markers	Variable durability	Vector/promoter optimization	[15,28]

The symbol → means ‘leads to’.

### 3.4. Emerging Perspectives: Microbiome Considerations

While hemophilia is defined by a deficiency of clotting factors, increasing attention has been directed toward putative systemic inflammatory and immune mechanisms that may influence disease expression and treatment outcomes. The gut microbiome, a central regulator of immune homeostasis, has emerged as a potential contributor to these processes [31]. However, this remains a hypothesis-generating area, and direct evidence in hemophilia is limited.

Chronic low-grade inflammation is frequently observed in patients with hemophilia, particularly in the context of recurrent joint bleeding and synovial damage [32]. Gut dysbiosis has been associated with systemic inflammatory activation through increased intestinal permeability and translocation of microbial products such as lipopolysaccharides (LPS) [33]. However, evidence linking these mechanisms specifically to hemophilia-related joint pathology remains limited. While such pathways may be relevant to systemic inflammation, their role in the development or progression of hemophilic arthropathy has not yet been established.

The microbiome may also influence immune tolerance mechanisms relevant to hemophilia treatment. Early-life microbiota composition has been implicated in shaping immune responses to therapeutic proteins, raising the hypothesis that microbiome-related factors could modulate the risk of inhibitor development [34,35,36].

Potential microbiome-targeted interventions, including dietary modification and probiotic strategies aimed at restoring microbial balance, are being explored in inflammatory and immune-mediated conditions [37,38,39]. Their relevance to hemophilia and gene therapy remains speculative and requires dedicated translational research.

At present, the microbiome should be considered a potential modifier of inflammatory tone and immune responsiveness rather than a confirmed determinant of clinical outcomes. Further studies are needed to determine whether these hypothesis-generating associations translate into clinically meaningful effects in hemophilia, including interactions with gene therapy–induced immune mechanisms (Table 2).

## 4. Clinical Translation of AAV Gene Therapy

### 4.1. Clinical Trial Outcomes and Approved Therapies

Over the past decade, clinical translation of AAV-mediated gene therapy has progressed rapidly in both hemophilia B and hemophilia A. Early clinical development focused on hemophilia B, as the smaller size of the factor IX gene facilitated efficient AAV packaging and contributed to more predictable expression profiles. Phase 1/2 studies demonstrated durable, stable FIX expression at therapeutically meaningful levels over long-term follow-up (3–5 years), accompanied by substantial reductions in annual bleeding rates (0.14) and bleeding cessation in a subset of patients, as shown in a phase 2b study of etranacogene dezaparvovec with sustained FIX activity (40–45%) maintained over 5 years [40]. The incorporation of high-activity FIX variants, such as FIX-Padua, has further enhanced therapeutic efficacy by enabling clinically meaningful FIX activity at lower expression levels.

Subsequent development in hemophilia A required the use of B-domain-deleted FVIII constructs to accommodate AAV genome capacity. Advances in vector engineering, promoter optimization, and dose selection have led to sustained FVIII expression and marked reductions in bleeding frequency in clinical studies [41,42,43,44].

Valoctocogene roxaparvovec (Roctavian), an AAV5-based gene therapy delivering a B-domain-deleted FVIII transgene to hepatocytes, has received regulatory approval in the United States and the European Union for adults with severe hemophilia A (Appendix A). A single administration can result in sustained FVIII expression, reduced annual bleeding rates, and discontinuation of routine prophylaxis in many patients [7]. Similarly, etranacogene dezaparvovec (Hemgenix), a liver-directed AAV-based therapy for hemophilia B utilizing the FIX-Padua variant, has demonstrated stable FIX activity, major reductions in bleeding episodes, and cessation of prophylaxis in a substantial proportion of treated individuals (Appendix A) [45].

### 4.2. Determinants of Clinical Response

From a clinical perspective, liver-directed expression is central to therapeutic success, as hepatocytes enable systemic secretion of clotting factors. However, dosing strategies require a balance between achieving adequate factor activity and minimizing immune-mediated hepatocellular responses, reflected by the association between higher vector doses and transaminase elevations. In addition, the high upfront cost of gene therapy, coupled with variability in reimbursement frameworks, contributes to substantial disparities in global access and may limit its widespread implementation in routine clinical practice. These disparities are especially evident in low- and middle-income countries, where structural, economic, and healthcare system constraints markedly limit equitable access to gene therapy.

### 4.3. Limitations and Barriers to Clinical Implementation

Despite these advances, several factors limit routine clinical implementation. Durability of expression remains variable, and a gradual decline in factor levels has been observed in some patients during extended follow-up, with FVIII activity decreasing from initial post-treatment of 40–60% to 15–20% over 2–7 years [46]. Because AAV administration induces durable anti-capsid immunity, current paradigms rely on a single-dose strategy, and loss of expression cannot be corrected through simple re-dosing. Additional barriers include pre-existing anti-AAV antibodies, high upfront treatment costs, restricted eligibility criteria, and the need for close monitoring of hepatic and immune-related adverse events. These limitations underscore that gene therapy, although transformative, remains a complex intervention requiring careful patient selection, structured follow-up, and long-term outcome monitoring.

Ongoing clinical research focuses on improved vector design, optimized dosing, and mitigation of immune responses to sustain expression. Despite robust phase 3 efficacy and safety data, real-world evidence remains limited. Preliminary data from post-commercialization cohorts and international registries are beginning to inform implementation, patient selection, and outcome variability [47]. Extended follow-up and real-world data are essential to define durability, long-term safety, and optimal integration into individualized hemophilia care (Table 3).

## 5. Individualized Management After Gene Therapy

### 5.1. Heterogeneity of Clinical Response and Expression Durability

Clinical responses to gene therapy in hemophilia are heterogeneous, with variability in the level and durability of transgene expression among treated individuals. Not all patients achieve optimal or sustained factor activity, and gradual decline in expression has been observed in a subset during long-term follow-up. These scenarios necessitate individualized post-gene therapy management strategies to maintain effective hemostatic control [7].

Although gene therapy aims to reduce or eliminate the need for replacement therapy, integration with established treatment modalities may be required in selected clinical situations. Conventional hemophilia care models, centered on prophylaxis and on-demand factor administration, remain relevant when endogenous factor levels are insufficient to ensure adequate bleed protection.

### 5.2. Post-Therapy Monitoring and Triggers for Intervention

Regular monitoring of factor activity after gene therapy is therefore essential, as early post-treatment expression does not necessarily predict long-term durability. Clinical decision-making should be guided not only by factor activity levels, but also by bleeding phenotype, as the relationship between transgene expression and bleeding outcomes may be non-linear. Declining factor levels may require implementation of supportive therapeutic strategies (Table 4) [45,48,49].

**Table 4:** Management strategies for suboptimal or non-durable response after gene therapy.

Clinical Situation/Strategy	Key Considerations	References
Resumption of prophylaxis	Reintroduction of factor replacement with dosing adjusted to residual endogenous factor activity	[45]
On-demand treatment	Factor administration for breakthrough bleeding episodes	[50]
Treatment optimization	Individualized modification of dose and infusion frequency based on partial endogenous factor production	[51]
Vector re-administration	Limited by pre-existing or treatment-induced anti-AAV immunity; investigational strategies include novel capsids, immune modulation and antibody-depleting approaches such as plasmapheresis or immunoadsorption, explored in the context of potential redosing protocols for valoctocogene roxaparvovec (Roctavian)	[48,49]
Transition to non-factor therapies	Use of rebalancing agents (e.g., emicizumab) as alternative prophylactic approaches independent of factor expression	[45,52,53]
Emerging gene-based strategies	Gene editing and alternative delivery platforms under investigation for future re-treatment possibilities	[48]

### 5.3. Management of Suboptimal or Declining Response

Suboptimal or non-durable responses to gene therapy represent an important area for systematic investigation. Comprehensive data collection on expression durability, anti-vector immune responses, bleeding outcomes, and subsequent therapeutic interventions is essential for improving long-term management [54]. The identification of predictive biomarkers may facilitate risk stratification and guide clinical decision-making, particularly in the context of declining transgene expression or consideration of re-treatment strategies. International collaboration through patient registries and research networks will be critical for generating sufficiently robust datasets to inform evidence-based care pathways. These efforts support the development of personalized strategies aimed at maintaining hemostatic protection and quality of life in patients with heterogeneous responses to gene therapy [54,55].

**Figure 3:**
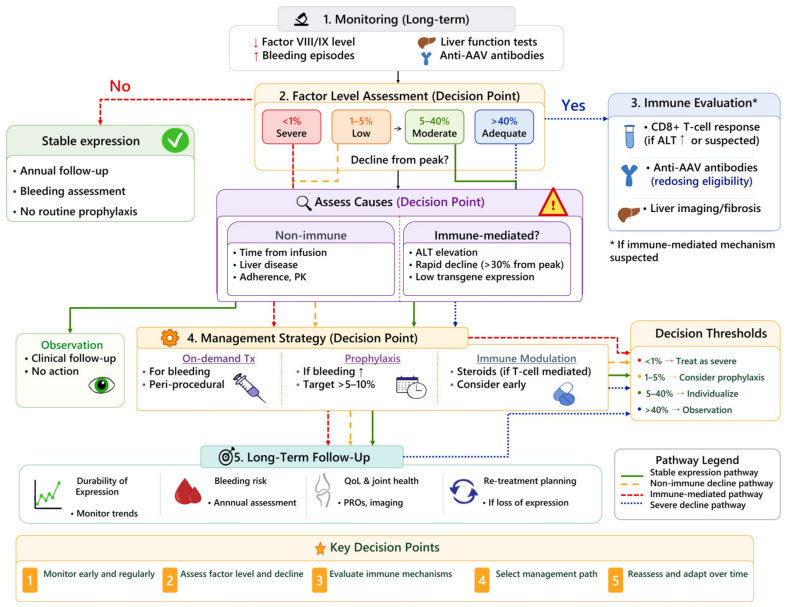
Clinical decision-making framework after gene therapy in hemophilia, integrating factor activity, bleeding phenotype, longitudinal expression dynamics, and patient-specific risk factors. The symbol “↑ indicates increase; ↓ indicates decrease.”

Clinical decision-making after gene therapy should integrate factor activity, bleeding phenotype, longitudinal expression dynamics, and patient-specific risk factors (Figure 3).

## 6. Quality of Life and Psychosocial Outcomes After Gene Therapy

Gene therapy in hemophilia is associated with clinically meaningful improvements in patient-reported outcomes, including reductions in pain, improved physical functioning, and enhanced overall quality of life (QoL) [56,57]. Reduced bleeding frequency and decreased treatment burden facilitate greater participation in daily activities, travel, and physical exercise.

However, transition from a long-standing chronic disease model to reduced treatment dependence introduces new psychosocial challenges. Patients accustomed to strict activity limitations may experience uncertainty in risk assessment and anxiety related to increased autonomy. Underestimation of bleeding risk and behavioral overcompensation have been described, particularly among younger individuals [32,58]. Structured education and counseling are therefore important components of post-gene therapy care.

Changes in treatment routines may also affect social dynamics within the hemophilia community. Reduced need for frequent clinical visits may decrease peer interaction and contribute to shifts in illness identity, which in some patients can lead to feelings of isolation [59]. Continued engagement through adapted support structures remains relevant despite clinical improvement. Holistic post-gene therapy care should therefore incorporate educational, psychological, and social support interventions alongside medical monitoring. Recognition of these psychosocial dimensions is essential for optimizing long-term outcomes and patient satisfaction following gene therapy (Table 5) [60,61].

**Table 5:** Domains of psychosocial and educational support following gene therapy in hemophilia.

Support Area	Clinical Relevance	References
Education	Helping patients understand the post-therapy health model, residual bleeding risk, and safe levels of activity	[60]
Psychological support	Supporting adaptation to reduced treatment burden and addressing anxiety related to changing disease perception	[60,61]
Social support	Maintaining peer interaction and community engagement despite reduced frequency of clinical visits	[60,62]
Individualized approach	Adjusting follow-up support according to patient-specific clinical status and psychosocial needs	[60]

## 7. Emerging Therapeutic Targets Beyond Factor Correction

Although factor replacement and gene transfer of FVIII or FIX remain central strategies in hemophilia management, increasing attention is directed toward therapeutic approaches that restore hemostatic balance without direct correction of the primary factor deficiency [63,64]. These strategies reflect a broader systems-based understanding of coagulation regulation and immune modulation.

### 7.1. Rebalancing Hemostasis

Rebalancing strategies target natural anticoagulant pathways to enhance thrombin generation independently of factor levels. Therapies reducing antithrombin activity and agents targeting tissue factor pathway inhibitor (TFPI) exemplify this approach. These strategies are particularly relevant for patients with inhibitors and illustrate a paradigm in which coagulation is restored through modulation of regulatory pathways rather than direct factor replacement [63,64,65,66].

### 7.2. Gene Editing Approaches

Emerging gene-based technologies extend beyond episomal gene addition. Genome editing strategies, including CRISPR/Cas9-mediated approaches, aim to achieve stable genomic modification of coagulation-related genes or regulators. These approaches may enable more controlled and durable correction while potentially reducing limitations related to vector persistence and immune responses. Notably, in vivo CRISPR-based gene editing for hemophilia B has recently entered early-phase human clinical trials, representing a key step toward durable genomic correction [67,68].

### 7.3. Immunomodulatory Strategies

Immune responses remain critical determinants of therapeutic outcomes in hemophilia. Strategies aimed at improving immune tolerance, such as modulation of adaptive immune pathways, control of anti-vector immunity, and induction of regulatory immune mechanisms represent an important area of investigation. These approaches may reduce inhibitor formation, stabilize transgene expression, and broaden eligibility for advanced therapeutic platforms [69,70].

### 7.4. Microbiome–Immune Interactions

The gut microbiome is increasingly recognized as a regulator of systemic immune tone and inflammatory balance. Alterations in microbial composition may affect immune responses and chronic inflammatory processes, potentially affecting disease expression and therapeutic responses. Although evidence in hemophilia remains limited, microbiome-related immune modulation represents a potential adjunct area for future research [32,33,34,35].

### 7.5. Closing Perspective

In aggregate, these emerging directions indicate a shift from isolated correction of coagulation factor deficiency toward integrated modulation of coagulation balance, immune regulation, and systemic inflammatory processes. Future therapeutic paradigms in hemophilia are therefore likely to combine hemostatic correction with targeted control of immunological mechanisms, reflecting an increasingly personalized and systems-oriented approach to disease management (Table 6).

**Table 6:** Emerging therapeutic directions beyond factor replacement.

Therapeutic Domain	Target Mechanism	Clinical Rationale	Development Stage	References
Hemostatic rebalancing	Antithrombin, TFPI modulation	Restore thrombin generation independent of FVIII/FIX	Clinical/advanced trials	[63,64,65,66]
Gene editing	Stable genomic modification	Potential long-term correction beyond episomal vectors	Preclinical–early translational	[67,68]
Immune modulation	Tolerance induction, anti-vector immunity control	Reduce inhibitors and improve durability of advanced therapies	Translational	[71,72]
Microbiome-related modulation	Regulation of systemic immune tone	Adjunct modulation of inflammation and immune responses	Exploratory	[32,33,34,35]

## 8. Summary

Gene therapy has reshaped the therapeutic landscape of hemophilia by enabling sustained endogenous production of the missing clotting factor following a single administration. This transition from lifelong replacement therapy toward durable biological correction represents a major advance in the management of this hereditary bleeding disorder.

Clinical studies have demonstrated marked reductions in bleeding frequency and treatment burden. However, variability in transgene expression, uncertainty regarding long-term durability, and immune-mediated limitations remain key challenges. These factors underscore the need to integrate gene therapy into structured care models incorporating patient selection, monitoring, and individualized post-therapy management, rather than viewing it as a definitive cure.

Immunological mechanisms are central determinants of therapeutic outcomes, influencing vector transduction efficiency, expression stability, and treatment eligibility. Understanding host–vector interactions and refining immunomodulatory strategies are therefore critical for improving durability and expanding clinical applicability.

Beyond direct factor correction, evolving therapeutic concepts aim to modulate coagulation balance, immune responses, and systemic inflammatory pathways. Rebalancing strategies, gene editing technologies, and tolerance-oriented approaches illustrate a broader shift toward systems-based management of hemophilia. Improvements in quality of life are substantial, yet psychosocial adaptation and ongoing support remain important components of comprehensive care.

The results should be interpreted in light of several limitations, including the absence of extended follow-up data, heterogeneity among the included studies, and the lack of randomized controlled trials in certain areas.

In conclusion, gene therapy represents a transition from replacement-based treatment to biologically integrated therapeutic paradigms. Continued translational research, longitudinal outcome data, and international collaboration will be essential to optimize durability, safety, and equitable access, ensuring that the full clinical potential of gene therapy can be realized.

## Figures and Tables

**Table 2:** Potential links between the gut microbiome and disease-modifying pathways in hemophilia.

Domain	Proposed Mechanism	Potential Clinical Relevance	Evidence Level	References
Systemic inflammation	Dysbiosis → intestinal permeability → LPS translocation	May contribute to chronic inflammatory milieu and joint pathology	Indirect/extrapolated	[33,37,38,39]
Immune tolerance	Microbiota shaping adaptive immune responses	Hypothesized impact on inhibitor risk	Exploratory	[35,37]
Immune regulation	Modulation of T-cell balance and cytokine profiles	Possible impact on treatment-related immune responses	Emerging	[36,39]
Therapeutic modulation	Diet, probiotics, microbiome-targeted strategies	Potential adjunctive support in inflammatory regulation	Investigational	[33,34,37]

The symbol → means ‘leads to’.

**Table 3:** Key challenges in clinical implementation of gene therapy in hemophilia.

Challenge	Key Considerations	References
Durability of expression	Variable long-term transgene expression; uncertainty regarding lifelong efficacy	[41]
Immune response	Pre-existing or treatment-induced anti-AAV antibodies; limited potential for re-administration	[41]
Costs	High upfront treatment costs (approximately $2.9 million for Roctavian and $3.5 million for Hemgenix per dose in the United States), with reimbursement remaining limited and uneven	[42,43]
Availability	Restricted eligibility (anti-AAV NAbs, liver disease, prior treatment); limited access in routine clinical practice	[43]
Safety profile	Transient liver enzyme elevations; need for monitoring and immunosuppressive management	[41]

## Data Availability

No new data were created or analyzed in this study. Data sharing is not applicable to this article.

## Appendix A

**Table A1:** Approved gene therapies for hemophilia and their mechanisms of action.

Active Substance	Brand Name	Therapy Type	Mechanism of Action	References
Valoctocogene roxaparvovec	Roctavian	Gene therapy used in severe hemophilia A treatment.	Uses an adeno-associated viral (AAV) vector to deliver a functional F8 gene into hepatocytes. The transduced liver cells begin producing factor VIII endogenously, which increases circulating FVIII levels and reduces bleeding episodes.	[7,32,58,71]
Etranacogene dezaparvovec	Hemgenix	Gene therapy used in moderately severe to severe hemophilia B treatment.	Employs an AAV vector carrying the Padua variant of the F9 gene, which encodes a hyperfunctional factor IX. Hepatocytes produce FIX continuously, providing sustained hemostatic activity.	[45,72]
Fidanacogene elaparvovec	Beqvez	Gene Therapy used in severe and moderately severe hemophilia B treatment.	Delivers a functional F9 gene using an AAV vector. Once inside hepatocytes, the introduced gene enables endogenous synthesis of factor IX, potentially maintaining therapeutic FIX levels long-term.	[16,73]
Emicizumab	Hemlibra	Non-factor therapy used in hemophilia A treatment.	A bispecific monoclonal antibody that simultaneously binds to the activated form of factor IX (FIXa) and factor X (FX). This mimics the cofactor function of factor VIII, enabling FX activation and thrombin generation even in the absence of FVIII.	[52,74]
Concizumab	Alhemo	Non-factor therapy used in hemophilia A and B treatment	A monoclonal antibody targeting tissue factor pathway inhibitor (TFPI). By inhibiting TFPI, it enhances the FX production via enhancing the tissue factor-initiated coagulation pathway, increasing thrombin generation and improving clot formation.	[75,76]
Marstacimab	Hympavzi	Non-factor therapy used in severe hemophilia A and B treatment	Binds to TFPI and inhibits its anticoagulant activity. This shifts the hemostatic balance toward clot formation by enhancing thrombin generation via the extrinsic coagulation pathway.	[66,77]
Fitusiran	Qfitlia	RNA-based therapy used in hemophilia A and B treatment	Small interfering RNA (siRNA) designed to silence hepatic synthesis of antithrombin. Reduced antithrombin levels increase thrombin generation, restoring hemostatic balance independently of FVIII or FIX levels.	[65,78]
Efanesoctocog alfa	Altuvoct	Replacement clotting factor therapy used in hemophilia A treatment	Recombinant FVIII molecule engineered to dissociate from von Willebrand factor and avoid rapid clearance. Structural modifications prolong circulating half-life, allowing sustained FVIII activity after infusion.	[79,80]
